# The Making of the Modern Practitioner

**Published:** 1912-03-09

**Authors:** 


### THE MAKING OF THE MODERN PRACTITIONER.

### Old and New Views on Asthma.

Every practitioner sooner or later has to deal with intractable cases of asthma; and much that he sees and hears in his intercourse with the sufferers from this distressing malady is knowledge of a kind which is quite foreign to his preconceived ideas formed in his student days from teachers and text-books. Face to face with the positive assurances of an educated and intelligent patient that this, that, or the other patent (and usually secret) remedy is the only treatment, the medical man is often sorely tempted to encourage, actively or passively, the self-drugging into which so many asthmatics seem eventually to drift.

At the outset it is necessary to insist on the phrase "true bronchial" asthma, since so many pulmonary complaints are denominated asthma by the unfortunate possessors of them. Chronic bronchitis, with or without emphysema; bronchiectasis; pulmonary tuberculosis even, both acute and chronic; and especially the pulmonary paroxysms and dyspnœa incidental to some forms of heart and kidney disease: all these are commonly spoken of by the public as asthma, and the resulting confusion only serves to intensify the difficulty of diagnosing and treating their own recurring troubles which asthmatics always get into. Because nitroglycerine may have given temporary relief to some plethoric sufferer from high tension and commencing cardiac dilatation, whose friends speak of his symptoms as "asthma," the same drug will be immoderately consumed by all the real asthmatics of the neighbourhood. Nor does the evil end there; for the morphine and cocaine habits find many victims among the ranks of the asthmatic; and the plight of those who thus add the horrors of a drug habit to those of habitual asthma is perhaps as lamentable as any that practitioners have to witness.

This true asthma has many characteristics which have been held to indicate that the nervous system takes a considerable part in its occurrence. The facts that certain smells or certain foods may excite a paroxysm, that certain localities suit some patients, while to others they are surely provocative of an attack; that some sufferers can live in comfort only at the seaside and others only in a town; that some get on at the top of a hill, whereas others can exist only at the bottom; these and many similar curious and inexplicable points in the clinical histories of patients have been taken as proof of some reflex or other nervous element in the ætiology of true asthma. Nevertheless, it cannot be said that any one of the current hypotheses concerning asthma has ever been demonstrated with any approach to scientific exactness. In general, the majority of clinicians subscribe to the hypothesis of a spasm of the muscular coats of the bronchioles. The weak points of this conception are that it fails to explain why the dyspnœa should be so essentially expiratory; and that it does not yield any indications of treatment which have proved of real practical value. Dr. J. B. Berkart has long held a different view; it is twenty-two years since the last issue of a book in which he suggested a bacterial pathology of asthma, but he has now lately published a new monograph wherein he again advances this view.* It is important to notice that this observer does not claim to have discovered a specific organism, nor even that there is any one microbe which alone will cause asthma. He suspects that a particular variety of streptococcus may have a good deal to do with the condition; but in general terms his position is that asthma is a symptom of an underlying sero-fibrinous bronchitis, and that to cure asthma it suffices to cure the local morbid lesions of the lungs which underlie the manifestations.

The full details of the treatment which Dr. Berkart advocates are set forth at length in his book. Oro-nasal hygiene, a correct dietary in small quantities at frequent intervals, open-air treatment and proper ventilation of all living-rooms, hot baths, the treatment of insomnia by some hypnotic such as paraldehyde, and various drugs for liquefying tenacious sputa and promoting their expectoration; this régime systematically pursued will, says the author, produce highly favourable results. For stramonium, lobelia, asthma powders and cigarettes, cocaine and atropine sprays, intranasal cauterisations, morphine, and frequent changes of climate before the chest is clear of physical signs: for all these he has nothing but the severest condemnation. Mont d'Or is mentioned but once, and even then somewhat slightingly; yet asthmatics, broadly speaking, agree that this resort really does them permanent good. In fact, all through Dr. Berkart will never listen to the fancies of patients. In obstinate cases he praises antidiphtheritic serum very highly, and apparently other authors have had similar successes with this agent.

For the acute paroxysm itself he gives sparing doses of morphine hypodermically, and oxygen. Though he mentions the risk of starting the morphia habit, he certainly fails to realise its full gravity. Of suprarenal extract he writes in a way which suggests that he has not tried it; yet its action is much more rapid than that of morphine, even if less certain. So striking and so rapid is the relief of the awful dyspnœa of a bad asthmatic paroxysm obtained by the injection of a few minims of this drug, that if Dr. Berkart had seen it for himself he would probably revise his condemnatory paragraphs concerning it. His main text, however, is that of an underlying bronchitis, and of asthma as a symptom of obstruction in the bronchioles due to the exudate thus formed. The fact that some of his views are contrary to ordinary beliefs and experience need not and must not prevent a very careful investigation into the truth or error of this hypothesis, which does at least provide a rational basis for treatment.

* *Bronchial Asthma.* By J. B. Berkart, M.D. Third edition. Oxford: University Press. Price 5s. net.

